# The Hospital. Nursing Section

**Published:** 1904-11-19

**Authors:** 


### The Hospital.

### Nursing Section.

Contributions for this Section of "THE HOSPITAL" should be addressed to the EDITOR, "THE HOSPITAL," NURSING SECTION, 28 & 29 Southampton Street, Strand, London, W.C.

No. 947.—VOL. XXXVII. *SATURDAY, NOVEMBER* 19, 1904.

### Notes on News from the Nursing World.

### QUEEN ALEXANDRA'S MILITARY NURSING SERVICE.

THE following new staff nurses have been appointed to Queen Alexandra's Imperial Military Nursing Service:—Miss A. E. Ansdell and Miss G. S. Jacob, posted to Alton; Miss L. Belcher and Miss E. McRobinson, posted to Woolwich; Miss C. T. Bilton, posted to Hounslow; Miss E. Eardley, posted to Yo k; Miss E. C. Fox, Miss F. N. Roberts, and Miss F. A. Smith, posted to the Connaught Hospital, Aldershot; Miss M. O'C. McCreery, serving at Dover; Miss M. E. Neville, serving at Malta; Miss D. J. Saunder, posted to the Royal Arsenal, Woolwich; Miss A. Willes, posted to Netley. Miss G. A. Magill, sister, and Miss K. Ward, staff nurse, have resigned their appointments. The appointments of Miss M. Steenson as sister, and of Miss W. M. Jay, Miss F. M. MacGregor, Miss M. MacGregor, Miss C. Mackay, and Miss B. F. Perkins as staff nurses, have been confirmed. Miss E. H. Hay, Miss E. J. M. Keene, Miss K. Pearse, Miss S. Smyth, Miss D. M. Taylor, and Miss A. L. Walker, staff nurses, have been promoted to be sisters. Sister A. Guthrie has been transferred from Malta to Station Hospital, Rochester Row, London; Sister E. J. M. Keene, from Woolwich to York; Sister S. B. Lanyon, from Hounslow to Woolwich; and Sister C. K. E. Steel, from Malta to the Connaught Hospital, Aldershot.

### MISS NIGHTINGALE AND THE SOUTH AUSTRALIAN NURSES.

Two addresses were sent from South Australia to Miss Florence Nightingale in honour of the attainment of her nursing jubilee. One was from the District Trained Nursing Society, and the nurses in the course of the address congratulated Miss Nightingale upon witnessing the jubilee of the beneficent system of trained nursing inaugurated by her on her arrival at Scutari, and expressed their belief that she would be gratified to know that the movement of which "this small token is one result," was started in connection with the District-Trained Nursing Society which originated in her own devoted services to humanity. The address is illustrated by representations of the Australian wattle, the Sturt pea, Lea Hurst, the Barrack Hospital at Scutari, St. Thomas's Hospital, and the Children's Hospital at Adelaide. The top centre is spanned by a ribbon bearing the words "She hath done what she could," and a scroll fills the bordering in the centre of the lower side in which is printed the famous stanza from Longfellow's "Santa Filomena." The other address was from the local branch of the Royal British Nurses' Association, and is in the form of a booklet bound in dark green leather. On the borders are representations of various local flowers, cleverly executed and charmingly arranged; Miss Nightingale's monogram is at the foot and over it are the words "Thy great work accomplished, rest in thine eventide." Miss Nightingale has requested the Agent-General for South Australia to convey her warmest thanks for the addresses to the nurses over the sea.

### THE FOREIGN NURSES WHO HAVE LEFT JAPAN.

THE member of the Order of Spanish American War Nurses who sent us the interesting account of the experiences of the Order in the Far East, which appeared in our issue of October 22nd, asks us to intimate that the nurses left the seat of the war a month later than was intended, and that the actual date of their departure was not September 20th but October 22nd. She adds:—"The members of the Order are at present enjoying their annual outing in St. Louis, including the Exhibition." For the sake of literal accuracy we gladly take the earliest opportunity of making this statement, but it does not in the least affect the position. However highly the services of the foreign nurses may have been appreciated by the Japanese authorities, the fact remains that when the heaviest of the work was coming, it was thought advisable to dispense with them.

### THE DUCHESS OF ALBANY AT THE EVELINA HOSPITAL.

ON Thursday last week the Duchess of Albany paid a private visit to the Evelina Hospital for Sick Children, in the Southwark Bridge Road. Her Royal Highness arrived at the hospital about 3.15 P.M., and was received by the chairman and staff of the hospital. The Duchess first ascended in the lift to the nurses' quarters on the third floor, and inspected the nurses' bedrooms and their sitting-room. Her Royal Highness was pleased to see that each nurse had a separate room, that the sitting room was airy and comfortable, and that the night nurses' quarters were separated from the rest by a glass door. She then proceeded through the surgical and medical wards, asking about every child's illness and saying a kind word to each. The Duchess next inspected the laundry, stores, and kitchen departments, and expressed her satisfaction with all that she saw. After visiting the out-patient department and partaking of tea in the matron's sitting-room, Her Royal Highness entered her name in the visitors' book, and left the hospital about 5 P.M., amid cheers from the children and others who had collected outside the entrance.

### QUEEN'S NURSES DESCRIBED AS USELESS.

The lady superintendent of the North Riding Rural Association and Cottage Hospital at Northallerton writes us, with reference to our "Note" of November 5th, that the organisation was once affiliated to Queen Victoria's Jubilee Institute, but that after the association had subscribed for some years "with no benefit to us," the authorities of the institute suddenly informed the managers of the association that the rural branch had been done away with a long time, and that unless they conformed to the rules of the institute they could not be affiliated to it—a very reasonable condition. The lady superintendent states that "this is quite impossible in rural districts like ours," and contends that it is far better for the association to be independent than "to be hampered by unsuitable rules." She also assures us that no nurse attached to the association has less than a year and seven months' training, that all are qualified at Glasgow Maternity Hospital, and that many have two years in a general hospital before they receive their maternity training. Finally, she asserts that "Queen's nurses are very good for district nursing in towns, but would be useless amongst the poor and farmers in the Yorkshire dales, where houses are miles apart." Seeing that Queen's nurses are doing excellent work in some of the most thinly-populated and remote corners of the kingdom, such as Achill Island in county Mayo, we are unable to understand why they should be considered useless amongst the poor of Yorkshire.

### OPENING OF THE HOME AT THE CANCER HOSPITAL.

The new home for the day staff at the Cancer Hospital, Brompton, was opened on Monday afternoon by Lady Ludlow, who, on arriving, was presented with a bouquet by the little daughter of the secretary, Mr. Howell. A short statement was then made by Lord Ludlow, president, who said that between £2,000 and £3,000 had been received in answer to the appeal issued in his name. The former inadequate housing of the nursing staff had, he continued, long been a source of anxiety to the committee, and the present building would, he hoped, amply justify the expenditure of some £4,000 to £5,000. In order to show exactly how the money had been expended, he proposed that the numerous visitors should adjourn to the home, which would be occupied for the first time by the nurses after the ceremony. In a hospital devoted to the treatment of cancer it was especially important that the committee should do all in its power for the nurses, whose work was arduous and heavy. When not on duty it was essential that they should be able to get away entirely from the scene of their labours. The chairman of the house committee welcomed Lady Ludlow and remarked that not only were the nurses made happy and comfortable by the completion of the home, but also the domestic staff, who, after being housed in the basement would now have suitable rooms on the top floor. He concluded by handing Lady Ludlow a silver key with which to open the building, and the company then proceeded to it The rooms, which were fully described in The Hospital of October 29th, were prettily decorated with plants and flowers. Yellow and white chrysanthemums and Neapolitan violets were conspicuous in the sitting rooms, which, with bright fires burning in the tiled hearths, pianos, tapestried sofas, easy chairs, and engravings on the walls, looked particularly cosy and inviting. The nurses, drawn up in a double row in the entrance, welcomed the visitors, and afterwards dispensed tea and coffee in the board-room.

### THE DELAYED NURSING ORDER.

At the annual meeting last week of the Poor-Law Unions Association of England and Wales, Miss Siddon, of Huddersfield, moved that a communication be sent to the Local Government Board asking for the early issue of the nursing Order consequent upon the report of the Departmental Committee, and the resolution was adopted. Whatever differences may exist respecting the nature of the recommendations of the Departmental Committee, there is absolute agreement as to the importance of some sort of pronouncement on the part of the Local Government Board. Even if the report is to be treated as so much waste paper, we may as well know authoritatively that the deliberations of the Committee are to be ignored.

### NURSING JEWISH PATIENTS AT THE LONDON HOSPITAL.

The four wards for Jewish patients at the London Hospital, which were opened on Monday, have been conveniently arranged with regard to the nursing. Each has its group of test-rooms, lavatories, and sinks. In the first, chiefly used by the students but accessible also for the nurses, who thus have the opportunity of gaining practical knowledge of testing, a small sink is fitted; there is a cupboard for apparatus, and slate shelves for bottles of specimens. Between each set of wards is a cosy sitting-room, which may be used by the sister if she wishes to remain within call while off duty, and for writing. These have cream-coloured walls with a dark-green dado, a picture rail, and an oak shelf running round the walls. They are furnished with writing-tables, rush-seated chairs, and a large screen. The floors are covered with linoleum. In the wards there are several innovations which are worthy of notice, as tending to save the nurses trouble. Electric light has been installed, and each bed is provided with a movable hand lamp, which can be either fixed or carried to the bedside. Linen curtains, of a chess-board pattern in black and white, are fitted to each bed, and these are drawn round by the nurse when necessary to secure privacy or quiet, the carrying of heavy screens being in this way largely avoided. All sheets, blankets, bed-jackets, etc, are stamped with the names of the wards. A special cupboard is provided for Passover crockery, which is only used on that occasion, and is of a different pattern and shape from that ordinarily employed. In addition to two sisters, each two wards will have its staff nurse, with three probationers. While the food must necessarily be prepared by a Jewish cook, it may be given, and the general nursing arrangements carried out, as usual, by Gentile nurses.

### A QUESTION OF TASTE.

On Tuesday a long paragraph appeared in a London daily contemporary about a new nurse who had lately entered a well-known London hospital as a paying probationer. She is described as "a slender girl, with soft wavy brown hair, neatly coiled under her cap, dark eyes, and a smiling mouth." Subsequently, she is alluded to as "one of the prettiest trio of sisters in society at the present time," and "the Earl's pretty daughter" is made to explain her position to the representative of the paper. There is no reason why the daughter of a peer should not become a probationer in one of the London hospitals, so long as she fulfils the necessary conditions. But there is every reason why her admission should not be paraded in the daily press, accompanied by a sketch of her personal appearance, by an account of the visits of her friends rolling up in their carriages in order to see her in her uniform, and by comments on the part of the new probationer respecting work of which a fortnight's experience does not qualify her to speak with authority.

### AN INFIRMARY WITHOUT A NIGHT NURSE.

In spite of the reiterated request of the medical officer of the Northallerton Workhouse, the Guardians have declined to sanction the appointment of a night nurse for the infirmary. The argument against the appointment appears to be that as the day nurse has only seven patients she could attend a special call at night. To this the medical officer rejoined that on a particular day, when he visited the infirmary he found one of the old women in the men's ward, and he asked, if this woman wandered about in the day time what would she do at night? Moreover, we presume that the number of patients may at any time be increased. At all events, one nurse should not be expected to take night duty in addition to day duty, however light the latter may be. We are satisfied that if the question is referred to the Local Government Board, as it should be, the attitude of the medical officer will be approved.

### IN THE INTERESTS OF THE TRAINING SCHOOL.

At the meeting of the Croydon Guardians, which we reported last week, the vice-chairman quoted a letter from the Metropolitan Asylums Board recognising the Croydon Union Infirmary as a training school for nurses, as a proof that no injustice is done to the probationers by not allowing the matron to sign the certificate. It is not surprising that when such an argument as this is employed, the majority of the guardians feel that their attitude is untenable, and we are in hopes that the infirmary committee will now realise the folly of prolonging the struggle. It is quite true, as one of the speakers said, that The Hospital has from the outset protested against the matron being deprived of her legitimate authority, and the probationers of the advantage of a properly signed certification, and we can also promise him that the subject will not be allowed to drop until the point is conceded. But it will be much more satisfactory for us to be able at an early date to congratulate the Croydon Guardians upon the reversal, by a unanimous vote, of a policy that cannot be maintained without detriment to a training school which should have before it a very considerable and important future.

### THE "MEDICAL REGISTER" SWINDLE.

It will be recollected that, in our issue for October 29th, we gave full details of an audacious attempt to deprive nurses of their hard-earned shillings by informing them that unless they had their names put down in the "Medical Register," they were liable to be thrown out of work. Among the victims we hear of three nurses who have been advised to take out a warrant of arrest against J. Howard and Co. and W. Morgan, whose names appear on the form of receipt. We should be glad if the offenders could be brought to book, but it is almost incredible that many persons can have been cheated by such a transparent swindle.

### THE STOCKPORT ASSOCIATION.

A feature of the report of the Stockport Sick Poor and Private Nursing Association, which was adopted at the annual meeting the other day, is that in the district department the income just covered the expenditure, while in the private department there was a deficiency of £25. The president of the association, in his speech, said that the public could have the assurance that their subscriptions would go entirely to the work of charity, and this is satisfactory so far as it goes. But from what source is the deficiency on the working of the private department for the year obtained? The earnings of private nurses should not be used in order to maintain a charity; and no portion whatever of the money contributed in order to provide nurses for the poor should, under any condition, be devoted to another purpose. When the two departments are combined under one management the temptation is not always resisted.

### A NURSE IN A MOTOR-CAR ACCIDENT.

In consequence of the sudden and serious illness of Lord Northbrook on Monday at midnight, his chauffeur was despatched from Stratton Park to Winchester for a doctor and nurse. He secured their services, and was returning with them about two o'clock on Tuesday morning, when the motor-car, descending a greasy hill just outside Winchester, crashed into the right bank and overturned, wrecking all the woodwork of the car and throwing the occupants out. The doctor's arm was broken, but the nurse was only bruised and shaken.

### NATIONAL SANATORIUM, BOURNEMOUTH.

The Committee of the Ladies' Household Linen Association in connection with the National Sanatorium, Bournemouth, held last week their first annual exhibition of the articles collected by the society in one of the—as yet—unfurnished wards. The institution was prettily decorated with flags and flowers, and tea was served to the visitors by the nurses in the women's corridor. The Ladies' Committee estimated the linen exhibited as worth at least £75; a very gratifying result for only seven months' work. Almost the whole of the linen and blankets for the new wards has already been supplied, while sufficient funds have been promised with which to purchase the articles which are still required.

### SUICIDE OF AN AMERICAN NURSE.

A very painful sensation was recently created in the Deaconess Home and Hospital, St. Joseph, Missouri, by the suicide of a nurse, who shot herself in the right temple and died early the next day without regaining consciousness. It is stated that fear lest she should become the victim of an incurable disease was the cause for the act.

### The Nursing Outlook.

"From magnanimity, all fear above;
From nobler recompense, above applause,
Which owes to man's short outlook all its charm."

### THE NURSING OF MENTAL CASES.

There is an interesting article on the training of mental nurses, by Dr. Urquhart, in the October number of the *Journal of Mental Science.* Dr. Urquhart thinks that the movement towards the nursing of male insane patients by female nurses is going too far; and is "a wrong to the male attendants." It is suggested that the lady nurse takes the highest posts and the highest fees, and calls in the male attendant when strength is needed, but leaves him no chance of rising in wages and responsibility. It would be sad indeed if the old wage-struggle between man and woman penetrated into the nursing world, but we do not believe this is probable.

Appended to Dr. Urquhart's paper is a statement by Mr. Bloomfield, who is himself a male nurse. He says:—

"It is a common idea that men do not make good nurses, but that is because very few men have had the opportunity of thorough training. My own experience is that, given equal conditions, male nurses can be trained to be quite as efficient as their female colleagues. Of course there are unsuitable men who enter on nursing, but I am convinced that if male attendants could be admitted to general hospitals for a course of nursing, the majority would be highly efficient, and could nurse any case intelligently."

It is to be hoped that Mr. Bloomfield will carefully watch all movements towards registration, particularly the two faulty Bills now the subject of inquiry by a committee of the House of Commons. There are too few good nurses of either sex so thoroughly interested in their profession as to take an intelligent interest in it as a whole, and male nurses most certainly have not properly organised means of making themselves heard. So that in discussing the merits and faults of male as opposed to female nursing, there are considerable difficulties.

The public as well as the profession should realise that if mental illness was taken early enough, and was approached with the definite aim and hope of cure, it would be found that the proportion of recoveries might be as high in mental cases as it is in physical cases. Instead of which the public have, so far, taken it for granted in mental cases that nothing active in the way of cure was possible; so the friends have shut the patients up, then in too many cases shown a desire to forget all about them, so that should they recover the greatest surprise is sometimes expressed. But active forms of ministering to the mind diseased are too often pooh-poohed by the followers of mechanical routine. Only a month ago, in the case of a young fellow whose delusion was that he had committed a crime, and who was in the first stages of disease, it was suggested by his relatives to send a priest to hear his confession and absolve him, and so try and give the mind a little ease. But the doctor declared "it will not have the slightest influence for good," and the quite harmless attempt at "suggestion" was never made. It would be greatly to the interests of the mentally afflicted if the friends could be induced to take steps to secure active medical treatment at the very commencement of the malady. Old prejudices should be eradicated, and every patient of this class should be held to be mentally ill, and so dealt with promptly.

Now it is the new school of medico-psychologists, who wish to approach brain disease as they would any other form of disease, who are largely in favour of the woman nurse; and this from the point of view that it is easier to get a woman trained in hospital methods, and that women are more bidable than men. There will ever remain a vast number of insane male cases which cannot, and should not, be nursed by ladies; and for our part we believe that there are also cases of physical disease which should always be nursed by their own sex. The wrong to the male attendant is not in setting a hospital nurse over him when necessary, but in not opening to him wider fields of hospital training. Miss Vernet, of the National Hospital, says: "I find the male nurses most helpful, and their care as regards cleanliness of patients, etc., is quite equal to that of the women nurses." But the National Hospital is practically the only training school for the men, and it is a "special," not a "general" hospital. It is one of the great needs of the present day that there should be some hospital or infirmary where male nurses could receive a training equal to that given to women at the best schools. Even such practice as the male attendants got in sick wards of an asylum is now going from them, as hospital nurses are put in charge. But this is not the root of the matter, and in so far as the male nurse is not trained, we hold the female nurse, for the sake of the patient, must be in charge. Men will never rush in great numbers into the nursing world: those who do enter it are probably well fitted for it, and should have every opportunity of learning; but where is their training school?

Meanwhile Dr. Urquhart says: "I do resent a certain condescension on the part of the trained hospital nurses"; so do we. Any want of tact which tends to set man against woman in the nursing world is to be sincerely regretted. Good nurses of either sex are rare enough, and there is room for them all.

### Lectures Upon the Nursing of Mental Diseases.

By Robert Jones, M.D.Lond., B.S., F.R.C.S.Eng., M.R.C.P.Lond., Resident Physician and Superintendent of the London County Asylum, Claybury.

LECTURE XXI. (*continued from page* 83.)

*Tuberculous ulceration of the intestine*, referred to under phthisis, is characterised by simple diarrhœa, and may be the only symptom of consumption of the lungs in insane persons, the expectoration being quietly swallowed and thus infecting the intestines and the mesenteric glands.

*Intestinal obstruction* is often fatal. It is caused most frequently by a hernia becoming strangulated; but it may be caused by a tumour pressing upon the intestine, or by the constriction of a band of fibres across the gut, by the growth of cancer involving the intestine in any part of its course; or in children, to one part of the intestine slipping into the tube or cavity of another part, like the inverted finger of a glove when pulled off the hand. This latter form is called *intussusception.* Obstruction may be due to one part of the intestine twisting upon itself through flatulence. From whatsoever cause the symptoms may arise; when constant vomiting, distension of the abdomen, pain or constipation occur, there is probably internal strangulation, and if relief is not forthcoming, death occurs in a few days.

*Appendicitis*, formerly called *typhlitis*, is inflammation of the *vermiform appendix*, which is attached to the commencing portion of the large intestine; the *cæcum*, situated in the lower part of the right side of the abdomen. There is pain and tenderness, feverishness and constipation with vomiting, and relief is to be obtained only by an operation which, to succeed, must be performed early. Therefore, it is of the utmost importance that the nurse should be able to observe symptoms so as to report them in time to save the life of the patient.

*Peritonitis* is inflammation of the delicate membrane lining the outer surface of the bowels. It occurs as the result of cold, disease, or injury, of a perforated gastric, typhoid or other ulcer, or as a complication of child-birth or puerperal fever. There is general abdominal pain, possibly not much temperature, but a weak and rapid pulse, the knees are drawn up, and the general pinched appearance is indicative of serious mischief.

*General Summary of Nurses' Duties.*—For all diseases of the intestinal tract, rest is necessary, and this can best be obtained by a partial or complete abstinence from food. This is especially necessary in cases of gastric ulcer or the hæmorrhage from it, and, for a time, all nourishment may have to be administered by the rectum. When food is taken by the stomach it may require *peptonising.* In most cases of gastric and intestinal trouble a simple dietary of milk with light farinaceous thickening, such as arrowroot, is alone allowed. This is done by mixing the peptonising powder or liquid with milk or beef tea at the body temperature and keeping thus for half an hour to an hour, then raising it to boiling point. Ice or iced water, soda or effervescing water, barley water and gruels are exceedingly useful to relieve thirst in acute cases. Purgatives must not be used for acute cases, and if there be constipation, a rectal enema is the safest first remedy. Every nurse must know how to give an enema, to note if it be retained and for how long. Hot applications, when ordered, should be light and the weight of the bed-clothes kept from the abdomen, as great care and tenderness is required in nursing cases of peritonitis.

*Diseases of the liver* are generally indicated by the presence of jaundice, but a slight degree of this may accompany pneumonia on the right side, when the movements of the diaphragm are impeded and the pumping action of this muscle upon the circulation of the bile is impaired. Jaundice may also occur with certain fevers, or in cases of obstruction of the bile duct from gall-stones, or from the extension of gastric catarrh to the orifice of the common bile-duct, which thus swells the mucous membrane and obstructs the flow of bile into the duodenum. There is usually much itching of the skin with jaundice, which can be relieved by alkaline baths, or by glycerine and water. The urine is highly coloured and the stools are pale. The sweat is yellow, and the linen may be stained thereby. There is often much loss of appetite and flesh, with sickness and vomiting. The nurse's duty in these cases is to pay especial attention to the bowels, that the "chair" is near the patient should he desire to get out of bed, and also to see that the sheets and bedding of the patient are comfortable, that he is kept clean and warm, and that he is as contented as his generally irritable disposition may permit.

*Disease of the Kidneys, or Renal Disease*—often called Bright's disease, as it was first described by old Dr. Bright, of Guy's Hospital—may be acute or chronic. The acute form may follow fevers, especially scarlet fever, diphtheria, and occasionally may accompany pregnancy. Neither is of common occurrence in asylum patients, although it might have been expected that the chronic form, which is called gouty or granular kidney, and is prone to follow drink and overwork, would be frequently met with. The cardinal or chief sign of kidney disease is the presence of *albumen* in the urine. The diseased kidneys permit the soluble nourishing constituents to pass from the blood into the urine, and there is anæmia, headache, palpitation, and probably dropsy. There is also a great tendency to hæmorrhage and apoplexy. The urine is scanty in the acute form; it is also highly coloured, of high specific gravity, and probably contains blood. In the chronic form it is copious, of a low specific gravity and light in colour. It is of great importance that the nurse should understand urine testing, as upon its condition depends the subsequent treatment. The urine should be collected for 24 hours and placed in a glass vessel; or, failing this, in a chamber, and allowed to stand. The (1) quantity is noted, (2) the colour and the amount of deposit observed; then (3) a strip of blue litmus paper is moistened with the urine, and if it turns red, the urine is of acid reaction, if it remains blue it is alkaline. (4) The little glass measure—the urinometer—is now allowed to float in it and the reading taken—probably between 1,010 and 1,030; (5) a small quantity is poured into a test-tube and boiled. If the deposit disappear and the urine remains clear then the deposit was urates; if, on the other hand, the deposit remains, or when there is no deposit, the boiled urine becomes cloudy, then the precipitate or cloud may be either albumen or phosphates. If, at this stage, two or three drops of strong nitric acid be added, the cloud of phosphates will disappear, but the albumen further clots and remains insoluble. Two other bodies, mucus and pus, may both be present in the urine, the former as a stringy or fleecy deposit, the latter yellowish or greenish-white. Blood in the urine gives it a smoky tint, and bile a yellow or mahogany colour. The nurse is only expected to test for albumen, to take the reaction (acid or alkaline), and to note the specific gravity of the urine. If she can accomplish this she saves the medical officer much time and may render very valuable assistance, whether in private nursing or in a public institution. The nursing treatment of all such cases is to take care to avoid cold by seeing that flannels are worn next to the skin, to apply vapour baths or medicines when directed and to see that there is always a free action of the bowels. Milk food with farinaceous thickening is generally prescribed as nourishment, and everything must be done to encourage a free flow of urine.

*Renal Colic*, lasting some hours, is accompanied by acute pain in the loins and is caused by the slow passing of a stone from the kidney to the bladder along the ureter. Hot baths and applications relieve the pain and spasm. The urine should be saved in such cases for the doctor to ascertain the nature of the deposit, which is an indication also of the nature of the stone.

*Diabetes*, a sad, trying, and invariably fatal disease among the insane, so far as my experience goes, is characterised by the excretion of a large quantity—usually several quarts—of urine in the course of 24 hours, by the presence of sugar in it, by constant thirst, a marked increase of appetite but with loss of flesh and power, and a gradually progressive emaciation. This disease is not infrequent among the insane. It aggravates the mental condition when it occurs after the onset of insanity and the treatment is, in the main, dietetic. All varieties of animal flesh, fish and fowl may be eaten, and milk in large quantities forms the staple article. All farinaceous food, *i.e.*, that containing starch and sugar, must be avoided. The patient is to be kept free from bedsores and should when the habits are defective be changed as soon as the occasion arises. These patients are very irritable and must be guarded against suicidal impulses. Much tact is needed to prevent irritation and imaginary offence. In my experience the sufferers have, previously to their insanity, been mostly very able-minded persons.

### Personal Experiences in a Salisbury Home.

BY A BRITISH NURSE.

"Well, I can but wish you well through your regimen of raw beef and hot-water sauce," said my good doctor and friend, to whom I had broken the fact, with some fear, that I was about to undergo a course of Salisbury diet. His words could scarcely be looked on in the light of a blessing on my experiment, but I was thankful to have received even a modified encouragement, for I had experienced so much cold disapprobation already from my lay friends that I was prepared for nothing short of a scolding from my doctor, whose dislike of dietetic fads was only too well known to me. However, dyspepsia is a form of misery which conquers all fears, and as it was a new one to me, I was fiercely determined to make all possible efforts to shake off its thraldom. A stray notice of a Salisbury Home and the memory of a bad case cured by the diet there prescribed were sufficient inducement to me to venture on the experiment; the character of my work was growing slack and my spirits were growing low, as all my previous efforts at shaking off my woes had been failures, though they had been conducted on the most orthodox lines. I decided, therefore, to try what other plans would do for me, and very shortly I was installed in a pleasant home, the head of which I found was a trained nurse, who was not only full of interest in her work, but anxious to make its principles understood by others.

### Treatment Commences.

My treatment, as I wished, began with the least possible delay, and very thorough in character I found it to be. The fact that I was confronted, on first wakening at 7 A.M., by a large jug of hot water, which had to be consumed slowly, taxed all my faith and enthusiasm. I had my "marching orders" from the doctor in charge of the Home, and knew very well that this jugful must be *sipped*; later on practice made me almost like hot water by the pint or quart, but at first the flesh was weakly, disappointing, and often I failed to finish my entire allowance—an omission which I was not allowed to consider in the light of a trifling matter of little import. After the hot water had been partly, or more often completely, absorbed, the time till breakfast at 8.30 was spent in quiet meditation; no getting-up, reading, or writing were permitted, and a very long time it seemed to me, but I had been much impressed by the desirability of quiescence both by the matron and by the doctor, and I tried to obey. There are, however, individual temperaments which fight against inaction of all kinds, mental and bodily, and the "rest hours" were to me all along a great trial, leading me to the view that enforced rest is as hard as enforced labour, though in a different degree. "Try to think of yourself as a cabbage," was the advice frequently given, and I did try—unfortunately, without success.

### My Breakfast.

Breakfast was brought to my bedside on a neat tray, and consisted of 3 oz. of minced beef in a small covered soup-plate, with 1 oz of very thin dry toast. *Cela tout*, and it was not by any means a "satisfying" meal. An hour's rest was compulsory after this curious repast, so I had ample time in which to try and imagine that I was not hungry! By about 10.30, if the day was at all fine, the patients were encouraged to go out; some had beef-tea or plasmon at this hour; milk I never saw during the whole time I was at the Home. At noon we assembled in the large dining-room engaged in sipping hot water, the long row of jugs and serious-faced patients made me dangerously inclined to laugh; I found a kindred spirit later on who was able to combine faith in the treatment with a commendable sense of humour, the latter sense I usually find suffers a sad decrease when its possessor becomes a "believer" in any new dietetic theory, and I therefore conclude the wish to convert others has a tendency to narrow the range of view. After the midday rite of hot-water drinking was over we went to our rooms for rest, some for an hour, others for half that time, and one or two were allowed to sit in the air but not to walk.

### Lunch and Dinner.

At 1.30 lunch was served; my portion was exactly the same as that at breakfast, except that I had an extra ½ oz. of minced meat. Some of the patients, who had already profited by the treatment, had quite large plates of mince, 8 to 10 oz., pudding also, or fish and carefully-cooked vegetables were allowed here and there. These were, however, the exceptions; as a rule, minced beef and crisp toast formed the basis of the meal; to that, as the cases advanced, a few additions were made, and very gradually the amount was increased. After lunch a rest was followed by a walk; then some patients were allowed to have one white of egg lightly poached; hot water was taken at five o'clock, rest from that till dinner at 6.30, which exactly resembled lunch as a rule; a small cup of tea or coffee was allowed to most patients after both meals, but as it was weak and without milk or sugar we were not tempted to excess in these stimulants. None of us suffered from thirst, as this had been quenched by the hot water an hour and a half before each of the principal meals. Rest after dinner, and a cup of plasmon or beef tea, massage, more hot water, and bed concluded the day.

### The Principles of the Treatment.

I will pause here to point out the principles which underlie this treatment, as thus I hope to make clearer what I

propose to say later on in summing up my view on the pros and cons which it shares with other forms of treatment. First, in cases of severe indigestion, of neurasthenia, rheumatism, or complications resulting from gouty conditions, the idea on which the Salisbury treatment is based aims at cleansing the system by the use of large quantities of distilled water, taken a sufficiently long period before meals to act as is suggested, "as an internal bath"; further the food is of a kind that is easily assimilated, and experimental variety is not attempted. It is considered that rest is very important both for the digestive organs and for the body as a whole, and that this can best be procured by the form of diet I have indicated. Sugar is seldom given except in cases which have advanced a good way on the road to health; the fermentation it so often sets up is guarded against, and for that reason milk is not approved as an article of diet as the sugar it contains is a dangerous factor according to Salisbury principles. For that and other reasons fruit is excluded in all the early stages of treatment; also vegetables and cereals. The objections to starchy foods are generally well-known, and if they are given to Salisbury patients they must be cooked for hours so that the starch may become thoroughly broken up. Many points in this treatment are now agreed on by specialists, but such was not the case a few years ago; even now very few people take the trouble to understand the principles underlying the Salisbury system, which may be summarised as an attempt to achieve a maximum amount of nourishment with a minimum of exertion to the digestive organs. I think that the smallness of the diet ordered is in some cases a distinct advantage, but in my own experience, although I at first gained in some ways, the actual hunger from which I suffered was very exhausting and increased, or seemed to increase, insomnia.

### Broken Rules.

One could not fail to be amused at some of the shifts to which poor humanity among the patients was put to obey orders outwardly, while occasionally indulging in forbidden luxuries. One poor gentleman, a model of correct behaviour in the Home, was one day found by the superintendent eating steadily through a plate of jam tarts in a confectioner's! This incident we did not hear at the time and so we wondered why Mr. Blank had of late grown so much more charitable to human frailty. How I envied his courage in eating those tarts, but I had been brought up on Puritan principles and so I found it impossible to do anything but to fast—though I admit I did so sourly and reluctantly. Truly, "it is one thing to be tempted and another thing to fall" and I have a pleasant consciousness that I was able to live through a summer of magnificent sunshine and strawberries, and never once to buy one, but I avoided passing fruitshops and looked the other way when I was compelled to do so. To some patients the consumption of large quantities of hot water became almost an impossible feat. Why the beautiful growing flowers in the conservatory should suddenly fade was a fact "wrapped in mystery" till someone discovered that a very rebellious girl refused to drink her jorum of water anywhere except in the immediate neighbourhood of some bulbs in enormous pots, and although she, I believe, went away cured, the poor tulips protested as strongly as they could against a hot water cure by a rapid death.

### Duration of Treatment.

I remained in the home for over eight weeks; my health was not so much benefited by the treatment at the end as at the beginning, although I continued the diet carefully for a further six weeks. I had then become painfully thin, and had lost about three stone in weight. This I knew was considered as quite a good sign, but in my case the wasting of muscles was a great drawback. I had massage for part of the time, and it seems to me an essential part if the treatment is to really succeed. I do not know that I was a very good case as I was very thin to begin with; in stout people the success seemed to me greater and more rapid. At the same time, although I have with advantage modified the strictness of the diet, I have kept to many of the Salisbury rules with distinct advantage. Hot water in moderation is excellent, but it should be taken a full hour before meals. Minced beef, if properly made, is also admirably adapted for many cases. I learnt to make both the mince and the toast, which are unexcelled outside a Salisbury Home; the latter is cut thin as for bread and butter, and is baked on a tin in a very hot oven. I can commend to nurses in private work the thorough mastering of these and other Salisbury recipes, their patients will be grateful for these and for the minced-beef cakes which are lightly grilled, according to special instructions. The diet is not an easy one to carry out at home; the ordinary cook objects to the time and the extreme care required in the mincing, etc., and I should think there was some opening for Salisbury nurses who could undertake the whole case and the cooking. In conclusion, I would say that although I think much of the system appeals to common sense, I would advise anyone thinking of trying it that they should consult a good doctor who knows their constitution in the first instance. It is distinctly not a treatment to attempt without good all-round advice as to its suitability for the individual case. Useful books for reference on the treatment, with recipes, have been written by Dr. Keightley and by Mrs. Elma Stuart. The system is named after Dr. Salisbury, an American physician, who made many experiments before propounding the diet.

### Examination Questions for Nurses in Our Colonies and Abroad Generally.

THE Question was as follows:—If called upon to nurse a case of scarlet fever in a house where there were other inmates, what steps should you take for the prevention of the spread of infection?

### First Prize.

Isolate patient at once, choosing two large, airy, sunny rooms in upper story, with access to a bath-room and water-closet, not used by other members of the household, if possible. Every unnecessary article of furniture, all hangings and rugs, I should remove before transferring patient, and protect the entrance to apartments with old sheets hung curtainwise, and kept moist with some disinfectant solution —1-20 carbolic acid, 1-1000 bi-chloride, or solution sulphate zinc and common salt (4 ozs. sulphate, 2 ozs. salt to one gallon hot water)—to prevent air from sick-room passing to rest of house as much as possible. The second room is mentioned because I should almost certainly have one member of the family quarantined with me, and we should eat and sleep in that room. Here, in Texas, should probably get our exercise on the upper "gallery" connected with our rooms and disconnected with rest of house, and the relative would communicate with other members of the household occasionally from this open-air point. I should see that she took care of herself in regard to disinfection at all times and get a good gargle from the doctor for her. Nothing should be used for or about patient which could not later be either boiled or destroyed. All soiled bed and body clothing used in both rooms should go at once into a tub of disinfectant solution for at least two hours, and then be boiled. Rags used as handkerchiefs and to receive other discharges would be burnt at once. All vessels used for excrementary matter should contain a disinfectant before, during, and after use, in addition to thorough scalding.

Should mop, not sweep floor with disinfectant, taking care not to make it too wet, dust with damp cloth. Flush closet well daily, after throwing in dry chloride lime. Water used for bathing and sponging should be disinfected before throwing away. Feeding utensils and dishes of others quarantined would not leave rooms, food being transferred from as few dishes as possible outside door.

During desquamation, oil eucalytus or carbolised oil as provided would be freely used, and such particles of skin as were collectable burnt.

Disinfectants in open dishes about the room are not far-reaching in their influence, yet are not to be despised. Saturated solution chloride lead is a good deodoriser and disinfectant. During convalescence would try and improvise amusements, that cherished toys might not be destroyed. Should save my doctor's time by having a large cotton garment and cap for him to don outside my curtain.

Recovery being complete, disinfecting baths and shampoos given, date of quarantine raising stated, would disinfect, boil, or destroy everything that had come within the infected area. Fumigate rooms with formaldehyde or sulphur, 2 lb. to 2,000 cubic feet space, in the presence of moisture, and recommend strongly after the room had been well aired that any plastering be renewed. Z.Z.Z.

### Second Prize.

In nursing a case of scarlet fever in a private house, a room must be selected as far removed as possible from those occupied by other members of the household. All unnecessary furniture, carpet, curtains, bed-hangings, etc., must be removed. A sheet saturated with a solution of bichloride of mercury (four tablets to water Oi.) or formalin (ʒi. to water Oi.) must be hung outside the door, and may be kept wet by means of a garden syringe.

No one must be admitted to the room but the doctor and the nurse. The latter must hold no unnecessary communication with members of the household, and must practise great personal cleanliness by constant use of soap and water and nail-brush, and careful disinfection.

A supply of crockery, etc., for her own and the patient's use should be kept in the room, any going from the room being previously scalded and soaked in a solution of formalin. Meals must be put outside the room on a tray, and fetched in by the nurse, all waste food being burnt.

Absolute cleanliness in all details, both with regard to her patient and the room, are essential.

When desquamation commences, the patient should be well washed all over, night and morning, with soap and water, and anointed with carbolised vaseline. All soiled bed-linen, patient's personal clothing, etc., should be immediately scalded and immersed in a solution of formalin, remaining there until sent to the laundry. Discharges from the eyes and nose must be received on pieces of old rag and burnt immediately.

In houses where sanitary arrangements are satisfactory, all excreta, waste water, etc., after disinfection, may be emptied in the water-closet. In other cases a deep hole must be dug some distance from the house and well—where it must be emptied and covered with earth. Great attention must be paid to the ventilation of the room, the window being kept continuously open, while guarding the patient carefully from draughts. The floor of the room must be washed daily with a weak solution of formalin; paint, furniture, etc., being carefully dusted with duster wrung out of same.

When desquamation is completed and the patient is declared free, before mixing with his friends he must be thoroughly bathed and sponged with a solution of formalin, the hair receiving especial attention. Fresh clothing must be provided. The nurse must take the same precautions with regard to herself.

In most cases the disinfection of the room is undertaken by the public authorities, but the nurse should be prepared to undertake it if necessary.

This is done most effectually, after sealing up all crevices and spreading out clothes which cannot be boiled, by inserting a bottle of formalin in the centre of a pan containing 1 lb. of sulphur, placing this in a basin containing a little water. The sulphur must then be ignited and the door immediately closed and sealed. The room must not be opened for 24 hours. Colonist.

### Virtues and Defects of the Papers.

"Z. Z. Z.," who has twice gained honourable mention in previous competitions, and who lives in Texas, sends in the best answer received this month, either from nurses at home or abroad. Her paper is practical and thoroughly to the point, and glances at the acute probability of having more than one patient at the same time, and that the different individuals should not be conveniently ill in the same degree and at the same stage. It is very interesting to have a side-light upon the domestic arrangements of other countries—certainly the Texan, "Upper Gallery" seems made on purpose for the satisfactory nursing of infectious diseases!

"Colonist" wins the second prize. Her idea of the use of the garden syringe is good. Other nurses have also discovered the utility of this humble implement.

### Honourable Mention.

This is gained by "Garde-Malades" and by "Hey, What," the latter has 26 erasures on her paper; so much carelessness creates a bad impression.

### Question for Next April.

In a case of fractured thigh put up with a long splint and extension, and in a case of hip disease treated with splint and extension, what precautions should you take for the prevention of splint sores?

### Notice to Nurses in the United Kingdom.

This question is only to be answered by nurses abroad, and not by those in England, Scotland, or Ireland. See rules at the end of the paper. The Examiner.

### Rules.

This week's competition is open only to nurses abroad. Answers must not exceed 500 words, and be written on one side of the paper only, without headings, divisions, or marginal notes. The pseudonym, as well as the proper name and address, must be written on the *same* paper, and *not on a separate sheet.* Papers may be sent in up to March 20th. All illustrations strictly prohibited. Failure to comply with these rules will disqualify the candidate for competition. Prizes will be awarded for the two best answers. Papers to be sent to "The Editor," with "Examination" written on the left hand corner of the envelope.

N.B.—The decision of the Examiner is final, and no correspondence on the subject can be entertained.

In addition to two prizes honourable mention cards will be awarded to those who have sent in exceptionally good papers.

### The Nurses' Bookshelf.

The Modern Nursing of Consumption. By Dr. Jane H. Walker. (London: The Scientific Press, Limited. Pp 47. 1904.)

Modern methods of treatment in phthisis have modified the nursing requirements to a greater extent than in most other ailments. Nursing in a consumptive sanatorium offers a career of usefulness to many women who are physically unable to stand the strain of ordinary hospital work, and it is perhaps only their misconception of the principles of open-air treatment which makes them hesitate to embark upon it. The author admits, however, that sanatorium duties are somewhat monotonous, and, at the same time, call for an attention to detail which renders them somewhat trying to nurses accustomed to more varied interests. While deprecating the employment of attendants without previous training, as is stated to be the custom in some foreign institutions, Dr. Walker considers six months in a hospital as sufficient training before entering a sanatorium, and appears to speak somewhat slightingly of the more fully trained nurse. This we regard as unfortunate, in so far as the supply of private nurses for consumptives must be largely drawn from those trained in sanatoria, and the judgment and sense of responsibility required in caring for a case of phthisis in a private house are scarcely likely to be adequately developed by six months in a hospital, even when backed by a certain amount of special experience. Hospital training means much more than the art of bed-making and the correct reading of a clinical thermometer. Apart from this, the reader will find much useful information as to the details of sanatorium treatment and their application to patients in private houses. To all who desire to inform themselves as to the nursing of consumptives the book may be thoroughly commended as a practical and trustworthy guide, and it may be depended on to supply that basis of exact knowledge which is the best security against a nurse's duties becoming a monotonous routine.

Working with the Hands. By Booker T. Washington. (Grant Richards. 6s.)

This is a sensible account of the Tuskegee Industrial Institute, U.S.A., for the training of young negroes. The author, himself a negro, writes with intelligence and lucidity of a work to which he has devoted a lifetime. The Institute at Tuskegee is formed on the lines of the Hampton, U.S.A., and has in its turn become the model for many others with the same aims in different parts of the United States and elsewhere. The book is profusely illustrated with photographs of members of both sexes at work in the shops or on the farm connected with the Institute. Readers interested in the future of the negro will find "Working with the Hands" an interesting and suggestive addition to books which have already appeared on the subject.

### Presentations.

St. Thomas's Hospital.—(By One of the Staff.)—It was with great regret that the nurses of St. Thomas's Hospital said "Good-bye" last week to Miss Lloyd-Still, who of late years has been in charge of the Home for Paying Patients. Although the responsible posts she has filled have been many, and wherever she has worked her influence has been great, it is perhaps as "Sister Charity" she will ever be remembered, for it was during the years she was in charge there that she first made herself beloved to all who came under her influence. Her knowledge of all things appertaining to nursing was indisputable, and her advice was constantly sought by those in doubt; but it is those, of whom in a big hospital there are many, who feel at times the want of sympathy and help, who have lost a true friend and will perhaps miss her most. She was a "sister" of whom St. Thomas's was indeed proud, and could, alas! ill be spared. Miss Lloyd-Still has left to become matron of the Brompton Hospital for Consumption, where she goes with the hearty good wishes of everyone connected with her old hospital. Before leaving she was presented, among other things, with silver candlesticks from the sisters and a silver rose-bowl from her old staff-nurses.

Chester Benevolent Institution.—On Tuesday last week several ladies of the committee and subscribers of the Chester Benevolent Institution met at 1 King's Buildings in order to present Miss Hazlem Davies with a testimonial, which consisted of a purse of gold, as a mark of esteem and appreciation of her services and work during the time she was matron. Letters were read from Katherine, Duchess of Westminster, and several ladies who were unable to be present. Miss Davies opened the Benevolent Institution in 1898.

### Appointments.

[No charge is made for announcements under this head, and we are always glad to receive, and publish, appointments. The information, to insure accuracy, should be sent from the nurses themselves, and we cannot undertake to correct official announcements which may happen to be inaccurate. It is essential that in all cases the school of training should be given.]

County Hospital, Newport, Monmouthshire.—Miss Marianne Hardy and Miss Edith Rigley have been appointed staff nurses. Miss Hardy was trained at the Macclesfield General Infirmary. Miss Rigley was trained at the Victoria Hospital, Blackpool, where she has since been staff and theatre nurse.

Hendon Sick Asylum.—Miss Ada Selina Morriss has been appointed charge-nurse. She was trained at the Brownlow Hill Infirmary, Liverpool, and the General Hospital, Maidstone. She has since been staff nurse at the Country Branch of the National Hospital at East Finchley, sister at the Poplar and Stepney Sick Asylum, and sister at the Homœopathic Hospital, Tunbridge Wells.

Hull Royal Infirmary.—Miss A. McLear has been appointed house sister. She was trained at the Royal Infirmary, Liverpool, and has since been sister at the General Hospital, Wolverhampton, and sister at the Royal Hospital, Sheffield.

Joyce Green Hospital, Dartford.—Miss Annie Unwin has been appointed charge nurse. She was trained at Shoreditch Infirmary, and has since been staff nurse and holiday sister.

North Lonsdale Hospital, Barrow.—Miss Ada Duckworth has been appointed theatre and casualty sister, and Miss Florence Poelin has been appointed sister of male wards. They were both trained at the Royal Infirmary, Liverpool, and at Queen Charlotte's Hospital, London.

Northampton General Hospital.—Miss Ada Berry has been appointed sister. She was trained at the Royal Hospital, Portsmouth.

St. Mark's Hospital, City Road, London.—Miss M. Innocent and Miss A. Cross have been appointed staff nurses. They were both trained at the Shoreditch Infirmary.

St. Mary (Islington) Infirmary.—Miss Lilian Piper has been appointed night superintendent of the female wards, Miss Annie Williams night superintendent of the male wards, and Miss Mary Simpson ward sister. Miss Piper was trained at Leeds General Hospital, and was subsequently charge nurse under the Metropolitan Asylums Board. Miss Williams was trained at the Birmingham General Hospital, and has since been charge nurse under the Metropolitan Asylums Board, ward sister at Islington Infirmary, and acting night superintendent. Miss Simpson was trained at University College Hospital, London, and has since been on the private staff of the Victoria Hospital, Chelsea.

Shoreditch Infirmary.—Miss Mary Savage has been appointed sister. She was trained at Bolton General Infirmary, and has since been charge nurse at the Park Hospital, Hither Green, private nurse at Blackheath, and sister at the General Infirmary, Burton-on-Trent.

Southport Convalescent Hospital.—Miss A. Duckworth has been appointed matron. She was trained at the Royal Infirmary, Preston, and has since been staff-nurse at Monkwearmouth Hospital, Sunderland, and at Leith Hospital. She has also been matron of the Infectious Diseases Hospital, Darwen, and nurse in charge of the male department of the Southport Convalescent Home.

Stourbridge and Halesowen Hospital.—Miss Ida Maud Moss has been appointed matron. She was trained at Tamworth Hospital and the City Infirmary, Birmingham. She has since been hospital and dispensary nurse at Market Drayton; sister at the Brook Hospital, London; nurse-matron at Stone Isolation Hospital; and matron at Pontardawe Isolation Hospital.

Union Infirmary, Birkenhead.—Miss Amy E. Longworth has been appointed temporary charge nurse. She was trained at Brownlow Hill Infirmary, Liverpool, and has since done private nursing.

### Everybody's Opinion.

[Correspondence on all subjects is invited, but we cannot in any way be responsible for the opinions expressed by our correspondents. No communication can be entertained if the name and address of the correspondent are not given as a guarantee of good faith, but not necessarily for publication. All correspondents should write on one side of the paper only.]

### GOOD NURSING.

"Sister D." writes: Referring to a letter in a recent issue, is it usual to give patients brandy to the amount of 1 oz. without water? I have always understood that this was a dangerous practice, and liable to bring on traumatic pharyngitis.

### THE SALARIES OF MIDWIVES.

"Certified" writes: At the meeting under the auspices of the Association for Promoting the Training and Supply of Midwives last week, Dr. Cullingworth seemed to imply that since the Act passed in 1902 there had been a lack of suitable midwives as candidates for the public posts. It seems to me that the reason is that the salaries offered are so small. Personally I (a registered midwife) would much prefer district to private nursing, but the salaries offered are utterly inadequate. The largest salary I have known to be offered to a midwife (who also had to be a three-years' certificated nurse) was £100. Is this any inducement to a well-educated gentlewoman? Needless to say, the salaries are more often about half that amount. What professional man would accept such an offer? The public seem to think that because a woman is a nurse, she should therefore philanthropically deny herself every comfort, to say nothing of luxury, for the rest of her life. If you look at the case from a business man's point of view, you see things in quite a different light. Consider the larger salary—the nurse has to find board and lodging, which will take about half the salary; then uniform and clothing, all recreation and travelling expenses, the annual holiday, charitable gifts (?), and to save for a possible day of need and provide for old age. Cannot the public manage to offer better salaries, and then they would have the choice of higher-class workers?

### NURSES FOR THE MIDDLE CLASSES.

"A Lover of District Nursing" writes: In the article "Nurses for the Middle Classes" a remark was made to the effect that some nurses were too sensitive to remain long at district nursing. After years of district nursing under several matrons I have come to the conclusion that many nurses are disappointed in the work simply because they have had to work under a matron who was not in sympathy with them. I was fortunate in my first district post to have a matron thoroughly in sympathy with both nurses and patients, and I was surprised to find how much can be done, not only in relieving the patient for the time, but in preventive work. The matron seemed to be in touch with all other agencies for the help of the poor, and whatever might be the trouble in a family, she could usually find some way of helping them. My next matron was altogether different and took no interest in the patients or their families outside the actual nursing. Her way of measuring work was by the number of visits paid, and often the nurses were sad and depressed on account of having to omit little attentions, which mean so much to a sick person, so as to get around to a large number of cases, some of which would not have suffered by having fewer visits. At that home we were constantly changing nurses as so many came to the conclusion that district nursing was too trying for them. I should like to say to those nurses who are discouraged in their first attempt, "Try again."

"An Irish Matron" writes: I was greatly interested in your article "Nurses for the Middle Classes," under the "Nursing Outlook," October 29th, and shall look with interest for further information as to the "homes" which you say it is proposed to start. I wonder if it would be possible for one or two suggestions to reach the unknown "benefactor" who proposes to start this scheme. In the first place, I think there is no question of the need of visiting nurses for the middle classes. As you say, the work would certainly commend itself to "elderly and sober nurses." Probably a great many of these have homes in London, or wish to make a home, with a mother or sister; they might also have some means of their own, and would prefer not to work the whole year round, and would yet be very sorry to give up nursing altogether. I would humbly suggest that to meet the needs of these "elderly" nurses, who are most likely to be the "steadfast workers and sober women" of your text, as well as the needs of the public, an association on the lines of the Nurses' Co-operation, 8 New Cavendish Street, of which I was a member for four years, would best meet the case. The nurses could then enjoy in their declining years all the pleasure and privacy of home life, and could also take a rest when necessary without losing their membership in the association. At the same time it would be quite possible to have a home at or near the office for those who have not homes of their own. I believe this work will commend itself to a great many women of real sterling character and skill, and if it is organised on broad lines should be a great success.

### INSPECTORSHIPS.

The honorary secretary of Dr. Barnardo's Home, writes:—I have just seen Sir William Chance's assurance that the "Inspector of boarded-out children who failed of her duty," and who is mentioned in your issue (on page 347) of September 24th, could not be one of the "careful and stringent lady inspectors" of the Local Government Board. That is as it may be, but why Sir William Chance should drag in our Society I do not know. It is a kind of insinuation that our lady inspectors are less "careful and stringent" than are those of the Local Government Board. I can only say that we have looked into this matter closely, and I am sure that the incident in question cannot refer to the children who are boarded-out by our Society, or to any of the lady inspectors employed by us. In fact no such incident ever happened within our knowledge. Moreover, we have been singularly free during the whole history of our boarding-out experience of any charge against foster-parents of harshness towards children, which says a great deal for the extraordinary care we have employed in selecting foster-parents and homes, and the still greater care of our continuous, unceasing supervision by qualified lady inspectors. The curious thing is that while Sir William Chance "girds" at us and appears to suggest an invidious comparison between our workers and those employed by the Local Government Board, I do not think I betray any great confidence if I add that it is within common knowledge that the Local Government Board have derived from time to time not a little information as to the best methods in dealing with boarded-out children from our example and experience. May I venture to add, in conclusion, that I think the time has now come when you ought to give us the name of the inspector referred to in your article of September 24th, or, if you cannot do that, at least tell us with what organisation she was connected; for otherwise it is clear that the "careful and stringent" lady inspectors of the Local Government Board, as well as those presumably less careful ladies of this association, and of yet another society (equally innocent), may feel that undeserved odium is resting upon them as being in some way connected with the lady inspector who so seriously "failed of her duty.

### Echoes from the Outside World.

### Movements of Royalty.

THE King and Queen, accompanied by Princess Victoria, arrived at Buckingham Palace on Monday afternoon from Sandringham. As the King walked to his carriage from St. Pancras Station he limped slightly, and it was stated that he had struck his foot while shooting recently. The injury, however, is trifling. There were large crowds inside and outside the station, and their Majesties were loudly cheered. The King subsequently held a Privy Council, at which he handed the Lord Chancellor the new Great Seal, a change in the design of the instrument having been rendered necessary by his Majesty's accession to the throne. The King and Queen, accompanied by Princess Victoria, then left town for Windsor to await the arrival of the King and Queen of Portugal.

### The King and Queen of Portugal.

GREAT interest is taken in the visit of the King and Queen of Portugal to England. Their Majesties arrived here on Tuesday afternoon, and travelled direct from Portsmouth to Windsor. At Portsmouth the Prince of Wales did the honours. At Windsor they were welcomed by King Edward and Queen Alexandra, and heartily cheered by the crowd. King Charles is a versatile monarch. He has written several translations of Shakespeare, is a sculptor and painter, and a man of genial and kindly ways. Queen Amelia, who was born at Twickenham, where her father, the Comte de Paris, resided in exile, received from the Pope the rare distinction of the Golden Rose, in recognition of her labours among the sick and poor. She has brought about the establishment of several hospitals in Portugal, and is a munificent supporter of them. After the conclusion of their visit to the King and Queen at Windsor, the headquarters of their Majesties will be at Buckingham Palace, but they are expected to pay several country visits before they return to Portugal, including one to the Duke and Duchess of Orleans at Wood Norton, Evesham.

### The War in the Far East.

IT was announced on Monday that several ships have reached Port Arthur within the past fortnight. The latest reports from the fortress are to the effect that the railway station, barracks, dockyard, and hospitals are all in ruins, having been destroyed by Japanese shells. The Russian troops sleep in the entrenchments. M. Perloff, the millionaire tea merchant of Moscow, has received a letter from Madame Stoessel, dated Port Arthur, October 24th, in which the wife of the Russian general in appealing for financial aid, says: "There are some soldiers here who have lost both arms, some who have lost either an arm or a leg, some without eyes, some wounded in the spine who will remain cripples throughout their lives. There are many, many unfortunate people."

### Mrs. Creighton on the Conference of Women Workers.

THE final sitting of the Conference of the National Union of Women Workers was held on Friday at York, and an address was delivered by Mrs. Creighton, summing up the work of the week. She said that though they were anxious to conquer new fields for women's energies, they had been reminded again and again during the conference that they had not got nearly enough women for the existing work. The whole mass of women were still rather dead, and the great evil of our young women was pleasure-seeking. They could not get the response from them that they wanted for work, because life in England for the comparatively well-to-do and healthy girl had been made so very pleasant that she wanted to enjoy herself and not to work. Mrs. Creighton confessed that she was getting tired of girls who wanted to be secretaries, when there was splendid work of nation-making to be done in our schools. In much of their talk during the conference, she wondered whether they had not been forgetting the home altogether. England must be built on the home and on the family, and while there was a great deal to be learned from Germany, we must avoid being municipalised in the same way. The trumpet-call to our women was to rise up and be more efficient, to do more work, and to do it better. She concluded with the warning that one great fault of women was that they were too fussy, and often lost all their charm when they took to work. It was essential to cultivate a spirit of repose.

### The Late Mr. Val Prinsep, R.A.

THE death of Mr. Val Prinsep, R.A., was announced on Saturday. Few of his friends knew that he was ill, and the end was quite unexpected. Mr. Prinsep, the son of a distinguished Indian Civil servant, was born in 1838, and began to study for the profession of an artist under Mr. G. F. Watts. His first canvas was hung at Burlington House in 1862, and, in every succeeding year he was an exhibitor at the Royal Academy. His first commission of importance was the painting of a picture of the great Indian Durbar, at which Lord Lytton proclaimed Queen Victoria Empress of India. This was exhibited in 1880. In 1879 he was made an Associate, and in 1894 he became R.A. He leaves a widow—to whom the King sent a message of condolence—and three boys, two of whom are at Eton.

### Two Picture Exhibitions.

THERE is a very varied show at the Winter Exhibition of the Royal Society of Painters in Water Colours. The landscapes in particular are mostly excellent. The President, Sir Ernest Waterlow, shows "A View of Wensley Dale," just green crops and grey skies, painted in that direct and pleasant style which makes the artist's productions refreshing to gaze upon. He contributes several others, all more or less noteworthy. "The Night Rack," by Mr. W. Matthew Hale, is a faithful representation of disturbed elements, and Mr. Napier Hemy has contributed some of his powerful sea-pieces, particularly "The Reef," "Rounding the Buoy," and "Setting the Spinnaker." "Autumn on the Tay," by Mr. D. T. Cameron, is treated with masterly breadth; and in "Upland Pastures" Mr. Tom Lloyd gives a glowing representation of real sunshine. "La Belle Dame Sans Merci," by Mr. Cadogan Cowper; "The Silken Cord," by Mr. Walter West; and "Istrian Boats, Venice," by Miss Montalba, should also be noted.—The collection of the Society of Portrait Painters at the New Gallery includes works of both living and dead artists, and it is a pleasure to see again such pictures as "The Late Sir Leslie Stephen" and "The Marchioness of Granby," by Mr. Watts—the latter one of the best pictures the veteran artist ever painted; the portrait of Philip Comyns Carr as a little boy, by Mr. Burne-Jones; and Mr. Sandys's crayon drawings of Lord Tennyson and Matthew Arnold. But the most remarkable portraits are those by Mr. Franz von Lenbach. Nothing he has done is finer than the two portraits of Bismarck, one painted when he was at the height of his power, and the other near the end, when weariness and age had sapped the statesman's indomitable energy. "The Emperor William I." and "Count von Moltke" are two more portraits by this gifted artist. Of modern pictures "The Hon. Mrs. Burrell," by Mr. John Lavery, is a beautiful study; and "The Daughter of A. Bailey, Esq.," by Mr. Shannon, and "The Countess of Clonmell," by Mr. Ellis Roberts, deserve special mention.

### A Book and its Story.

### MR. NORRIS'S NEW NOVEL.*

MR. NORRIS is a veteran novelist, to whose pages we turn in confidence that he will give us a good story, with well-drawn characters and incidents which, if not too tragic or dramatic, are at least natural, and lacking in the morbid elements which a newer school finds so necessary to the success of its creations.

In "Nigel's Vocation" no character is superfluous. Each one has its place, and works out its destiny after much apparent uncertainty as to its ultimate fate in a manner which illustrates how important the art of suspension and diversion is in sustaining the interest of the reader in the development of the plot. The motive of Mr. Norris's present novel is a rather unusual one. Nigel Scarth—in religion, Brother Anselm—has, when a young man, fled from the temptations of a world whose attractions were too strong for his powers of resistance, to the seclusion of monastic life. He is found on the opening page, standing in the grounds of Lew Abbey surveying the result of a day's work. So far his experience of monastic discipline had extended only to a novitiate of two years. The description of him at work in the kitchen garden of the monastery is a suggestive one. "Brother Anselm drove his hoe into the soft ground, relinquished his grip for a moment, and sighed somewhat wearily, although he was not tired, in a physical sense, by a long day's work at trenching, manuring, and clearing away the weeds, which a spell of mild spring weather had brought to the surface by thousands. That season of the year, as everybody knows, is wont to produce stir and unrest throughout the realm of creation, including its lords, and even monks, whose whole life is a defiance of natural laws, must not expect to be altogether exempt from the operation of these. . . . Brother Anselm had seen little of the world, and had tasted enough of its pleasures and temptations to dread them as his deadliest foes; all he asked now was to be allowed to take final vows, priests vows, and—thus expugnably protected—to work his way by the grace of God, to those eternal vows which cannot deceive or disappoint . . . " That Brother Anselm was sincere in his aspirations no one, when the story of his life is read, can doubt. The cleverness of the author's delineations consists in the skilful way in which it represents the man's realisation of his own weakness. In this the strength is shown, which comes out forcibly as occasions occur for its expression. The old walls of the grey abbey stand out as a foil to the figure of the young postulant awaiting with impatience the day when, his years of probation ended, he would become a full member of the Order. "The ancient abbey behind him, restored and re-inhabited after centuries of disuse, and looking down upon the rich, pleasant Western country, of which the community now owned some fifty acres, was the symbol of their final victory over an evil sensual world by a patient self-denying army, in the ranks of which he was a recruit. His trouble was that he was still only a recruit, still upon his trial, still reminded by constant mortifications, reproofs, penances, that his submission was not recognised for the complete unchangeable fact that his heart knew it to be." Brother Anselm's reflections are interrupted unexpectedly by the appearance of an old lay brother, who comes stepping across the freshly-dug beds of the vegetable garden where Brother Anselm is at work, to summon him to the Lord Abbot. He asked no questions. Experience had taught him to refrain from doing so, also to control any expression of anxiety or curiosity which such a command might naturally excite. Upon entering the reception-room of the monastery, set apart for visitors, he finds, in addition to the Abbot, two other persons awaiting him. The Abbot, "a clean-shaven, emaciated, hairless old man, with a kind face and sunken blue eyes, made a slight motion with one hand towards one of them, but did not speak. The stranger rose slowly. He was big, gaunt, somewhat round-shouldered, had a hook nose, a long upper lip, and iron-grey hair. Brother Anselm noticed. He said, "You do not know me, Nigel, although I presume that you must have heard of me. I am your Uncle Robert." Brother Anselm (or Nigel Scarth) had known little or nothing of his father's relations. They had never had any interest in him as the son of a not very creditable member of the family. His Uncle Robert was in consequence unknown, except by name, to him. But the striking and urbane person by whom he was accompanied, was not so entirely a stranger. Before he had entered the monastery Nigel had had many interviews with him and remonstrances, in his capacity of family lawyer to the Scarth family, on the reckless disposal of his small fortune. Mr. Linklater on this occasion had a pleasanter task to perform. He was there as the bearer of the will of Nigel's late uncle, in which, subject to certain conditions, he was given a life-interest in his two-landed estates and the greater part of the income arising from the personal property. The first condition of succession was that Nigel must at once leave the monastery and take up his position as a country squire; the second, that the property, in the event of his marriage, would pass to his son, if he had one; the third (only if he were a Protestant) made the life interest cease if any time he should become a monk.

The prospect of returning to the world had no immediate attractions to Nigel, who knew his own instability of character only too well. He did not crave for riches now, but he faltered a little in his decision to reject what he would formerly have called his good fortune, but which now he regarded as a temptation of the Evil One, as he was moved by the consideration that it would be in his power, temporally at least, to assist the Church into which he had been received, by pecuniary offerings of some magnitude. Nigel turned, naturally, to the Abbot in his difficulty. At first his impulse had been to reject at once the offer which was made to him under the terms of the will; but "the faded blue eyes of that mild old man, which had on more than one previous occasion pierced the innermost recesses of his soul, made him pause." With another long sigh, he said, "If I were to accept, it would be for the sake of the Church." The Abbot replied that as a Catholic layman he would, if leading a good life, serve the Church well, but that the interests of the Church were far less concerned in the matter than his own, adding that he knew he would accept unless forbidden, and that he could not undertake the responsibility of making the decision for him. But Nigel declares that he will not accept the offer to return to the world. "I shall refuse, father." "So be it, my son. We shall rejoice to keep you with us, just as we should rejoice to welcome you back. . . ." "You would receive me again, father, if—if I were to find the world, the flesh, and the devil too powerful for me?" "Undoubtedly. However, I have a higher opinion of your courage than you have." Here we leave our hero at the threshold of a new experience. His conduct under the changed conditions of his life, his disentanglements from the complications which surround it, and the final return to the shelter of the monastery, is graphically and cleverly worked out.

* "Nigel's Vocation." By W. E. Norris. Methuen. 6s.

### Notes and Queries.

**FOR REGULATIONS SEE PAGE 12.**

*Homes.*

(49) Can you kindly tell me of a home where an epileptic girl aged 21 could be received? She is quite helpless and would require a great deal of attention. Her parents could afford to pay a small sum weekly.—*P. N. P.*

The Meath Home of Comfort, Westbrook, Godalming, and St. Luke's Home for Epileptic Churchwomen, 36 Parkwood Road, Bournemouth, seem the most likely to admit the case.

Will you kindly tell me if there is a free home where a little girl who suffers from bronchitis could be received for a year? Somersetshire preferred.—*Nurse B.*

Write and ask if she is eligible for the Home for Sick Children, Belmont House, Winchcombe Street, Cheltenham.

Can you tell me of a home in Hastings or St. Leonards where an elderly lady suffering from rheumatism and weakness could go?—*Nurse M. S.*

Possibly the Hastings and St. Leonard's Home for Invalid Gentlewomen, Catherine House, Church Road, St. Leonard's-on-Sea, might suit her.

*Sanatoria.*

(50) Is there any sanatorium where a lady could be received for a weekly sum of 15s.? She is suffering from mental hysteria, but her case is considered unsuitable for the county asylum.—*E. M.*

Write to the Holloway Sanatorium Hospital for the Insane, St. Anne's Heath, Virginia Water.

Can you give me the name of hospitals and homes in Edinburgh, or tell me how I may procure them?—*H. J.*

"Burdett's Hospitals and Charities" gives a full list.

I should be glad if you can tell me of a home where a schoolmaster, aged 48, could be received. He was discharged incurable two years ago from an open-air sanatorium. It is a very sad case, and his friends can only afford to pay about 6s. a week.—*District Nurse.*

Write to the Secretary of St. Barnabas Home, 9 Lloyd Street, W.C.; St. Michael's Home, Cheddar; or St. Barnabas Home, Brocket Hall, Torquay.

*Book.*

(51) Will you kindly tell me of a good book on elementary nursing and cases of emergency? A book somewhat fuller than those of the St. John's Ambulance Association, and suitable to take abroad.

"First Aid to the Injured and Management of the Sick," by E. J. Lawless, M.D. Published by the Scientific Press.

*Qualifications of a Training School.*

(52) What is the smallest number of beds in a cottage hospital which enables its governing body to give certificates to its nurses? Is there any fixed rule?—*Hon. Sec.*

The authorities of any hospital or home are at liberty to give certificates to the nurses, however few the beds.

*South Africa.*

(53) Will you kindly tell me if there is a nursing home in or near Grahamstown, South Africa?—*Nurse R.*

The matron of the Albany General Hospital, Grahamstown, may be able to give you the information. Enclose stamps for reply.

*Abroad.*

(54) Will you kindly let me know to whom I should apply for work in the South of France? Please give me the names of some of the homes there?—*K. S.*

The Nice Nursing Institute, Villa Désambrois, Nice, might be able to employ you. See "Burdett's Hospitals and Charities" for full list.

Will you kindly give me the names of nursing homes or hospitals in large towns in Germany where fully trained English nurses would be likely to obtain employment (Dresden preferred)?—*Dresden.*

We doubt whether there is any opening in Germany for English nurses.

**Handbooks for Nurses.**

"A Handbook for Nurses." By Dr. J. K. Watson. 5s. net; 5s. 4d. post free.

"A Complete Handbook of Midwifery." By Dr. J. K. Watson. 6s. net; 6s. 4d. post free.

"The Nurses' Pronouncing Dictionary." 2s. post free.

"Elementary Anatomy and Surgery for Nurses." Price 2s. 6d.

How to Become a Nurse: "The Nursing Profession." 2s. 4d. post free.

The above may be obtained of all booksellers or of The Scientific Press, Limited, 28 & 29 Southampton Street, Strand, London, W.C.

### For Reading to the Sick.

SOME THOUGHTS ON PRAYER.

Go up and watch the new-born rill
Just trickling from its mossy bed,
Streaking the heath-clad hill
With a bright emerald thread.

Canst thou her bold career foretell,
What rocks she shall o'erleap or rend,
How far in ocean's swell
Her freshening billows send?

Even so, the course of prayer who knows?
It springs in silence where it will,
Springs out of sight, and flows
At first a lonely rill:

But streams shall meet it by and by
From thousand sympathetic hearts,
Together swelling high
Their chant of many parts.

*Keble.*

There is a Jacob's ladder set up on earth through the mediation of the Redeemer, the top of which reaches to Heaven; and the Lord, standing above it, at the opened gate of everlasting life, says to the soul of each disciple, "Come up hither." Moreover, He has furnished us with angel's wings to help us in our upward journey, the wings being good desires. But to what purpose are the wings if we will not use them to rise?—*E. M. Goulburn.*

There can be no Christian life where there is a prayerless life; as impossible is it to live naturally without breath, as to live supernaturally without prayer. It is because prayer is so all-important that we find it so difficult. We ought to expect it to be difficult just because it is essential; and it will be difficult, being essential, because of the mystery of temptation.—*G. Body.*

Do not only pray for temporal blessings: pray for these objects, but pray still more for an increase of patience, for more faith, and more hope, and more submission to the will of God It may be better for you or others that you should not obtain the temporal favours you ask; but He will assuredly grant you the prayer for spiritual blessings. Life without prayer is not the life for which God created man; it is not the life of a rational being: it is an earthly, an animal life, in which the supernatural is omitted, in which the supernatural end and the supernatural means are wholly lost sight of.—*George Porter.*

O Thou, Whose love is not confined to temples made with hands, enlarge my heart to worship Thee. Help me to see Thee where men see only the world, to hear Thee where men hear only the voices of the crowd.—*George Matheson.*

Nor fear lest sympathy should fail:
Hast thou not seen, in night-hours drear,
When racking thoughts the heart assail,
The glimmering stars by turns appear,
And from the eternal home above
With silent news of mercy steal?
So Angels pause on tasks of love,
To look where sorrowing sinners kneel.

*Keble.*

